# Long-term effects of severe acute malnutrition on lung function in Malawian children: a cohort study

**DOI:** 10.1183/13993003.01301-2016

**Published:** 2017-04-06

**Authors:** Natasha Lelijveld, Marko Kerac, Andrew Seal, Emmanuel Chimwezi, Jonathan C. Wells, Robert S. Heyderman, Moffat J. Nyirenda, Janet Stocks, Jane Kirkby

**Affiliations:** 1 Institute for Global Health, University College London, London, UK; 2 Malawi-Liverpool Wellcome Trust Clinical Research Programme, University of Malawi College of Medicine, Blantyre, Malawi; 3 Department of Population Health, London School of Hygiene and Tropical Medicine, London, UK; 4 Leonard Cheshire Disability and Inclusive Development Centre, Dept of Epidemiology and Child Health, University College London, London, UK; 5 Childhood Nutrition Research Centre, UCL Great Ormond Street, Institute of Child Health, University College London, London, UK; 6 Division of Infection and Immunity, University College London, London, UK; 7 Respiratory, Critical Care and Anaesthesia section in III, UCL Great Ormond Street, Institute of Child Health, University College London, London, UK

## Abstract

Early nutritional insults may increase risk of adult lung disease. We aimed to quantify the impact of severe acute malnutrition (SAM) on spirometric outcomes 7 years post-treatment and explore predictors of impaired lung function.

Spirometry and pulse oximetry were assessed in 237 Malawian children (median age: 9.3 years) who had been treated for SAM and compared with sibling and age/sex-matched community controls. Spirometry results were expressed as z-scores based on Global Lung Function Initiative reference data for the African–American population.

Forced expiratory volume in 1 s (FEV_1_) and forced vital capacity (FVC) were low in all groups (mean FEV_1_ z-score: −0.47 for cases, −0.48 for siblings, −0.34 for community controls; mean FVC z-score: −0.32, −0.38, and −0.15 respectively). There were no differences in spirometric or oximetry outcomes between SAM survivors and controls. Leg length was shorter in SAM survivors but inter-group sitting heights were similar. HIV positive status or female sex was associated with poorer FEV_1_, by 0.55 and 0.31 z-scores, respectively.

SAM in early childhood was not associated with subsequent reduced lung function compared to local controls. Preservation of sitting height and compromised leg length suggest “thrifty” or “lung-sparing” growth. Female sex and HIV positive status were identified as potentially high-risk groups.

## Abstract 2

**Despite stunting, survivors of acute malnutrition do not have worse lung function than controls** http://ow.ly/2nvH308LroU

## Introduction

The Developmental Origins of Health and Disease (DOHaD) theory proposes that environmental exposures (such as nutritional insults), during critical time “windows” in early life have long-term implications for adult health, particularly non-communicable diseases (NCDs) [1]. Respiratory illness is no exception; there is evidence that early nutritional insults, both prenatally and during early infancy, result in higher risk of chronic obstructive pulmonary disease (COPD) and asthma [2, 3]. The exact mechanisms behind DOHaD are not currently known, but it is hypothesised that disruption of lung development, particularly alveologenesis, could lead to chronic lung disease in adulthood, particularly when compounded by adverse environmental factors [4, 5]. Since the majority of alveologenesis is thought to occur before the age of 2 years [6], nutritional insults during this period could result in long-term impairments. In particular, severe acute malnutrition (SAM), which is most prevalent in the first 2 years of life, might have an important biological link to long-term respiratory disease.

Worldwide, SAM affects more than 19 million children under 5 years [7, 8] and is defined as low weight-for-height (below −3 z-scores using the World Health Organization (WHO) growth standards) or a low mid-upper-arm circumference (<115 mm) or presence of oedematous malnutrition (a glossary of nutrition terms and definitions can be found in the supplementary material) [9]. Although some studies have considered the effects of chronic undernutrition (also known as stunting) [10, 11] and micronutrients [12, 13] on lung function, there is very little evidence regarding the long-term effects of SAM on lung function.

Understanding whether SAM during infancy and childhood has lasting effects on lung function could facilitate development of interventions aiming to curb the growing burden of NCDs in developing countries and improve survival and long-term outcomes post-SAM. The aim of this study was to quantify the long-term (7-year) effects of SAM on spirometry outcomes, as well as explore predictors of poor lung function, including severity of stunting and wasting, presence of oedema at admission, exposure to household cooking smoke, sex and HIV, in a cohort of Malawian SAM survivors [14, 15].

## Materials and methods

### Study design

This was a longitudinal cohort study that prospectively followed-up ex-SAM “case children”. We estimated that we would locate and recruit 300 case children plus one sibling and one community control per case. This was sufficient for detecting differences in lung function between groups equivalent to 0.5 z-scores (>90% power, 5% significance), generally thought to be the cut-off for clinical significance [16].

Ethical approval for this study was granted by Malawi College of Medicine Research and Ethics Committee (COMREC) (reference P02/13/1342) and University College London Research Ethics Committee (reference 4683/001). Besides lung function, other outcomes were also studied: more complete details of the cohort, as well as additional methods and results have been described elsewhere [17, 18].

### Study setting and subjects

The cohort originally included all patients admitted to the nutrition ward for treatment of SAM at the Queen Elizabeth Central Hospital, Blantyre, Malawi, from July 12, 2006 to March 9, 2007 (1024 children). The median age of the children at admission was 21.5 months (interquartile range, 15–32 months). Results of survival and anthropometry at the baseline study and the 1-year follow-up have been described previously [14, 15]. 47% (477/1024) of the original cohort were known to be alive 1 year post-discharge; these children formed the “case group” for this study. The sibling control was that closest in age to the case child, between the ages of 6 and 15.9 years. Those younger than 6 years were not eligible for spirometry testing, due to the difficulty in obtaining high-quality spirometry in very young children under field conditions. The community control was defined as a child living in the same community, of the same sex, and within 12 months of age as the case child. This community control was randomly selected by spinning a bottle at the case child's home to select a random direction, then enquiring door-to-door to find the first eligible child. Children who had ever been treated for acute malnutrition were excluded from the control groups. Informed written consent was obtained from the child's parent or guardian; assent was required from the children themselves. Possible sources of missing data included cases who could not be located, those who agreed to participate but did not attend the hospital appointment (more often community controls) and those who failed to achieve spirometry data of acceptable quality.

### Variables

Primary lung function outcomes were forced expiratory volume in 1 s (FEV_1_), forced vital capacity (FVC) and the FEV_1_/FVC ratio expressed as z-scores, based on the Global Lung Function Initiative (GLI) reference equations for the African–American population, which adjust for standing height, age, sex and ethnicity [16]. Spirometry testing took place in our study room at the hospital using the Easy on-PC spirometer (NDD medical, Zurich, Switzerland), operated by N. Lelijveld or E. Chimwezi, and one of three trained data collectors. Quality control of acceptable spirometry traces was undertaken weekly by N. Lelijveld. J. Kirkby, who was blinded to the study groups, checked 40% of results. The criteria for acceptability and repeatability of the spirometry curves were based on American Thoracic Society/European Respiratory Society recommendations modified slightly for children [19, 20].

Duplicate measures by independent data collectors were made for height and sitting height to the nearest 0.1 cm (Leicester height stadiometer, HM-250P; Marsden Weighing Group, Rotherham, UK), and weight to the nearest 0.1 kg (MS4202L; Marsden group, Rotherham, UK), following the WHO method [21]. Weight-for-age (WAZ), height-for-age (HAZ) and body mass index (BMI)-for-age (BAZ) z-scores were calculated using WHO 2007 growth standards [22]. HIV status was established from results in health passports; if unknown, an HIV test was offered by a trained counsellor, following Malawi national protocol (using Determine HIV-1/2 (Abbott Laboratories, Irving, TX, USA) and Uni-Gold HIV (Trinity Biotech PLC, Bray, Ireland) tests). Puberty was recorded as a binary variable, as reported by the participant or guardian (onset of menarche in girls, voice change in boys). As birthweight was rarely recorded, mothers were asked whether their child was “small” or “normal/large” at birth, during the baseline data collection; this follows a method widely used in Demographic and Health Surveys (DHS) (http://dhsprogram.com/). Pulse oximetry was used to measure oxygen saturation (*S*_pO_2__) using the NONIN PalmSAT 2500 device (Nonin Medical Inc., Plymouth, MN, USA) during the iStep exercise test (incremental step test) developed by UCL Institute of Child Health [23]. Body composition, including lean mass, was measured using bioelectrical impedance analysis (BIA) (Quadscan 4000; Bodystat Ltd, Douglas, UK). A questionnaire detailing contra-indications for spirometry and potential confounding factors, including cold symptoms, asthma, cooking fuel use, socioeconomic circumstances (SEC) and history of respiratory illness was also administered. These questions were based on the “American Thoracic Society's ATS-DLD-78C respiratory questionnaire”, UCL's “SLIC” study questionnaire and Malawi DHS [24–26]. History of respiratory illness was assessed by parental reporting. Exposure to household cooking smoke was defined as those reporting use of solid fuels for cooking inside the home.

### Analysis

**FIGURE 1:**
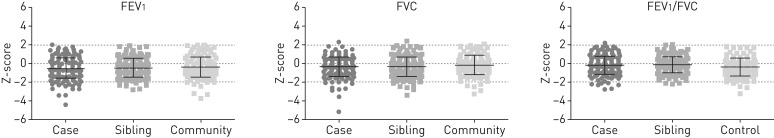
Lung function results for cases, siblings and community controls. Solid line with error bars represent mean±sd. Dashed lines indicate the limits of normality as per Global Lung Function Initiative (GLI) spirometry reference data for the African–American population (*i.e.* mean (0) ±1.96 z-scores). There were no statistically significant differences in any of the spirometry outcomes among the three groups. Most results fall within the normal range; however, mean forced expiratory volume in 1 s (FEV_1_) and forced vital capacity (FVC) were lower than predicted by the GLI reference for all three groups.

Primary analysis was based on all children with technically acceptable (grade A–C) spirometry results. Those with grade D results (*i.e.* not repeatable) [19] and those with reported current upper respiratory (cold) symptoms were also included as they were evenly spread across the sample and made no significant difference to the final conclusions [27]. Basic demographic data for each study group as well as those cases lost to follow-up are presented (table 1). Data for the three main spirometric outcomes and exercise test outcomes were examined by allocation group (figure 1) and statistical differences assessed using simple and multivariable linear regression models (table 2 and table S1). One model was used for each outcome where the predictor was coded zero for cases, one for siblings and two for community controls. HIV status, age, sex, SEC derived from asset scores using DHS questions [26], and puberty were included as *a priori* potential confounders. For spirometric outcomes, sitting height as a percentage of standing height and leg length were also explored as potential confounders in further analysis. The difference in odds of completing the exercise test between allocation groups was calculated using multivariable logistic regression.

**TABLE 1:** Demographic characteristics for the three study groups and those lost to follow-up

**Basic demographics**	**Cases**	**Sibling controls**	**Community controls**	**Cases lost to follow-up**
**Subjects n**	237	164	131	190
**Age (range) years**	9.3 (7.6–15.3)	11.5 (4.6–15.6)	9.1 (5.2–15.1)	8 (7–19)
**Males**	128 (54)	76 (46)	68 (52)	108 (57)
**Birth order median (interquartile range)**	2 (1–4)	2 (2–3)	2 (1–3)	2 (1–3)
**Started puberty**	7 (3)	12 (7)	6 (5)	NA
**SEC (asset quintile)**				
1 (poorest)	50 (21)	36 (22)	22 (17)	NA
5 (richest)	44 (19)	28 (17)	28 (21)	NA
**HIV**				
Seropositive	65 (28)	5 (3)	3 (2)	44 (23)
Seronegative	155 (65)	100 (61)	75 (57)	121 (64)
Status unknown	17 (7)	59 (36)	53 (40)	25 (13)
**Height-for-age z-score**	−1.8±1.2	−1.5±1.2	−1.3±1.1	NA
**Weight-for-age z-score**	−1.6±1.0	−1.4±1.0	−1.2±1.0	NA
**BMI-for-age z-score**	−0.8±0.9	−0.8±0.9	−0.7±0.9	NA
**Sitting height %**	52.2±1.5	51.8±1.7	51.8±1.5	NA
**Sitting height cm**	65.4±4.3	68.2±7.1	66.0±4.7	NA
**Leg length cm**	59.9±5.5	63.0±9.6	61.6±6.0	NA
**Lean mass index**	9.02±1.1	9.20±1.0	9.12±1.0	NA
**Reported indoor biofuel use (wood/charcoal)**	37 (16)	∼16%	18 (14)	NA
**Reported indoor tobacco use**	31 (13)	∼13%	19 (15)	NA
**History of TB**	11 (5)	1 (0.6)	1 (0.8)	4/173 (2)
**History of pneumonia admissions**	7 (3)	13 (8)	7 (5)	NA
**Ever admitted to hospital (except SAM)**	47 (20)	43 (26)	38 (29)	NA

Results are presented as n (%) or mean±sd, unless otherwise indicated. Height-for-age z-score is based on World Health Organization 2007 growth standards. Lean mass index is calculated from impedance results of bioelectrical impedance analysis and height: a higher value implies more lean mass. Sitting height %=sitting height/standing height×100. History of tuberculosis (TB), pneumonia and hospital admission were self-reported. Cases lost to follow-up are those who were admitted with severe acute malnutrition (SAM) in the original cohort but could not be subsequently located. “∼” for sibling controls indicates that this was not measured but assumed to be the same as for cases with whom they shared a household. NA: not applicable. SEC: socioeconomic circumstances; BMI: body mass index.

**TABLE 2:** Results of simple and multivariable linear regression analysis for spirometry outcomes across the three study groups

**Cases (n=201)**	**Mean±sd**	**Sibling (n=143)**			**Community (n=121)**		
		**Mean±sd**	**Difference case–sibling**		**Mean (sd)**	**Difference case–community**	
			**Unadjusted (95% CI)**	**Adjusted (95% CI)**		**Unadjusted (95% CI)**	**Adjusted (95% CI)**
**FEV_1_ z-score**	−0.47±1.1	−0.48±1.0	0.02 (−0.2 to 0.2)	0.13 (−0.2 to 0.4)	−0.34±1.1	−0.13 (−0.4 to 0.1)	−0.02 (−0.3 to 0.2)
**FVC z-score**	−0.32±1.0	−0.38±1.1	0.06 (−0.2 to 0.3)	0.20 (−0.0 to 0.5)	−0.15±1.1	−0.17 (−0.4 to 0.1)	−0.05 (−0.3 to 0.2)
**FEV_1_/FVC z-score**	−0.21±0.9	−0.15±0.9	−0.06 (−0.3 to 0.1)	−0.10 (−0.3 to 0.1)	−0.37±1.0	0.16 (−0.1 to 0.4)	0.15 (−0.1 to 0.4)

Adjusted differences include HIV status, socioeconomic circumstances and puberty. When including sitting height % in the model, all p-values remain >0.05. FEV_1_: forced expiratory volume in 1 s; FVC: forced vital capacity.

For our secondary analysis, HIV status, SEC, sex, history of pneumonia, history of TB, cooking smoke exposure and body composition (lean/fat mass) were examined for their association with spirometry outcomes across the entire sample using linear regression. A sex-stratified analysis on cooking smoke exposure was also conducted to try to explain significant differences in spirometry found between the sexes.

We also used linear regression to explore predictive factors of poor spirometry outcomes in the case group only. Namely, differences associated with severity of wasting and stunting at admission, presence of oedema at admission, HIV status, sex, estimated birth size and age at admission (months) were calculated after adjusting for HIV, SEC and puberty (table 3). For all analyses, a p-value of <0.05 was considered to indicate statistical significance.

**TABLE 3:** Results of linear regression analysis comparing effects of potential predictors of poor long-term spirometric lung function in severe acute malnutrition survivors (case group only)

**Potential predictors**	**Adjusted (95% CI) difference z-score FEV**_**1**_	**Adjusted (95% CI) difference z-score FVC**	**Adjusted (95% CI) difference z-score FEV**_**1**_**/FVC**
**Severely stunted at admission (HAZ <−3) (n=92 *versus* 105)**	0.02 (−0.3 to 0.3)	0.25 (−0.1 to 0.6)	−0.17 (−0.5 to 0.1)
**Severely underweight at admission (WAZ ≤−4) (n=92 *versus* 106)**	−0.07 (−0.4 to 0.2)	0.20 (−0.1 to 0.5)	−0.46* (−0.7 to −0.2)
**Oedema at admission (n=164 *versus* 34)**	0.05 (−0.4 to 0.5)	0.01 (−0.4 to 0.4)	0.25 (−0.1 to 0.6)
**HIV positive *versus* negative (n=53 *versus* 133)**	−0.58* (−0.9 to −0.3)	−0.49* (−0.8 to −0.2)	−0.23 (−0.5 to 0.0)
**Poorest *versus* richest SEC (n=118 *versus* 76)**	−0.27 (−0.06 to 0.0)	−0.22 (−0.5 to 0.1)	0.01 (−0.3 to 0.3)
**Males *versus* females (n=108 *versus* 91)**	0.32* (0.0 to 0.6)	0.27 (−0.0 to 0.6)	0.06 (−0.2 to 0.3)
**Low *versus* normal birth size (n=18 *versus* 176)**	−0.02 (−0.6 to 0.5)	−0.04 (−0.6 to 0.5)	−0.06 (−0.6 to 0.4)
**Original admission age ≤2 years *versus* >2 years (n=106 *versus* 93)**	−0.22 (−0.5 to 0.1)	−0.21 (−0.5 to 0.1)	−0.02 (−0.3 to 0.3)

*: indicates p<0.05. Results are adjusted for HIV status, socioeconomic circumstances (SEC) and puberty. n=201 for all cases with spirometry results; n presented for each predictor indicates small numbers of missing data for each variable. FEV_1_: forced expiratory volume in 1 s; FVC: forced vital capacity; HAZ: height-for-age z-score; WAZ: weight-for-age z-score.

## Results

**FIGURE 2:**
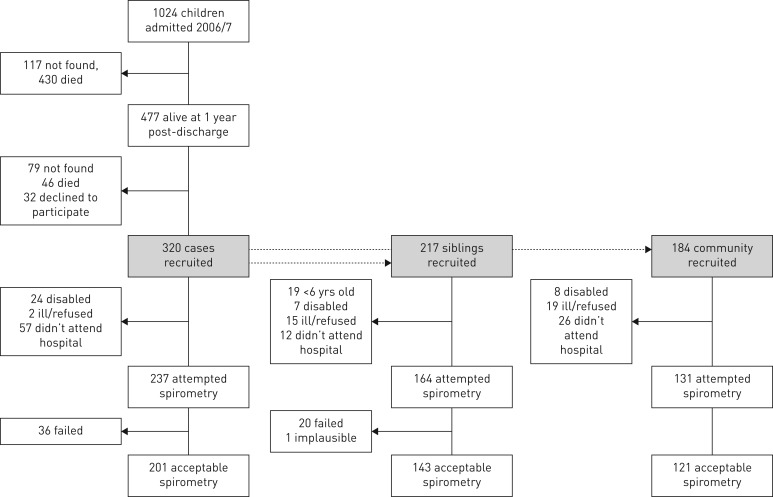
Recruitment flow diagram for spirometry results. Recruitment of sibling and community control only commenced at the time of 7-year follow-up; hence, their recruitment history starts after that of cases, who were enrolled at admission.

In all, 398 out of 477 (83%) case children families were contacted; 32 declined to participate, 46 case children had died, and 320 case children were investigated. The study included 721 children in total (320 cases, 217 sibling controls and 184 community controls). Those who attempted spirometry and the final number of technically acceptable results are described in figure 2. The proportion of acceptable results was similar across the study groups (85% for cases, 87% for siblings and 92% for community controls). Although the study aimed to recruit one of each type of control per case, not all cases had an eligible sibling, and some community controls dropped out of the study prior to the hospital appointment. Exploration of important demographic and health characteristics for the three study groups and those cases lost to follow-up indicated that HIV was a key potential confounder in the case group (table 1). All three groups had mean height, weight, and BMI z-scores below global reference norms (table 1); however, cases were significantly more stunted than siblings (adjusted mean (95% CI) height difference: −0.2 (−0.4 to −0.0) z-scores) and community controls (−0.4 (−0.6 to −0.2) z-scores) (see supplementary material for definition of stunting). Further details of growth and survival outcomes are published elsewhere [18]. There was no statistically significant difference in chest circumference, chest depth, or sitting height between cases and controls, but cases had a significantly shorter leg length than either sibling (adjusted difference −1.43 cm, 95% CI −2.3 to −0.5) or community controls (adjusted difference: −1.97 cm, 95% CI −3.0 to −1.0).

### Lung function differences across study groups

Although most subjects had spirometry results within the normal range (*i.e.* within 0±2 z-scores; figure 1), mean FEV_1_ was significantly lower than predicted in all the groups (by an average of 0.47 z-scores) [16]. Similarly, albeit slightly smaller than for FEV_1_, deficits were also observed for FVC. There were however no significant differences in FEV_1_, FVC or FEV_1_/FVC z-scores between cases and either type of control (table 2; figure 1). This remained true when adjusting for sitting height or leg length in the regression model. Additionally, there were no significant differences in *S*_pO_2__ either at rest or during the iStep exercise test between the study groups (table S1), although cases were less likely to complete the exercise test than controls (log odds of completing the test adjusted for age, sex, HIV status and SEC: −0.34 (−0.7; 0.0) compared to siblings; −0.53 (−0.9; −0.1) compared to community controls).

### Predictors of lung function in the whole sample

In the entire sample, FEV_1_ and FVC z-scores were significantly lower for HIV-positive children than HIV-negative children (0.5 and 0.4 z-scores lower respectively, unadjusted p<0.01 for both) with no significant difference in the FEV_1_/FVC z-score (p=0.2). There was no difference in spirometry outcomes for those with a previous pneumonia admission, whereas in those with a history of TB (n=13; note small sample size), FEV_1_ and FVC were lower by mean (95% CI) 0.91 (−1.5 to −0.3) and 1.07 (−1.7 to −0.4) z-scores, respectively (unadjusted p<0.001 for both).

Although there was no difference in FEV_1_/FVC, FEV_1_ and FVC z-scores were both significantly lower in girls than boys for the sample as a whole (unadjusted difference (95% CI): −0.28 (−0.5 to −0.1) z-scores, p=0.004, and −0.25 (−0.4 to −0.1) z-scores, p=0.012, respectively). These statistically significant deficits remained after adjusting for HIV status, SEC and puberty (as well as after adjusting for sitting height percentage and/or leg length). There was no significant difference in age, wealth or HIV status between the sexes for the sample as a whole, although boys were slightly but statistically significantly more stunted than girls (unadjusted difference (95% CI) HAZ: −0.2 (−0.4 to −0.04)).

There were no statistically significant differences in spirometry outcomes for those exposed to tobacco smoke at home or for those who lived in homes that cooked with biofuel inside (wood/charcoal). Sex-stratified analysis of the whole sample found no statistically significant difference in FEV_1_ or FVC z-scores for males exposed and unexposed to household cooking smoke. However, FEV_1_ was 0.5 (95% CI −0.0 to 1.0) z-scores lower (p=0.059) in females exposed to household smoke than in unexposed females (borderline statistical significance; adjusted for HIV and SEC). There were no significant differences in FVC or FEV_1_/FVC z-scores.

Lean mass index as calculated from BIA was positively associated with FEV_1_ and FVC z-scores (after adjusting for HIV, SEC, age, sex and puberty: 0.26, p<0.0001 for FEV_1_ z-score; and 0.28, p<0.0001 for FVC z-score). SAM (*i.e.* study group) was not included as a potential confounder in this section of analysis, as it was found to have no significant effect on lung function in this sample.

### Predictors of lung function in the case group only

We also explored whether factors which differed between the cases at admission could predict poor spirometry outcomes at 7 years post-discharge (table 3). There was no significant difference in spirometry outcomes according to the degree of wasting, degree of stunting, age at admission or presence of oedema at the time of admission, after adjusting for HIV status, SEC and puberty. Reported birth size (*i.e.* mother's report of her child being “normal or big” *versus* “small”) also had no association with long-term spirometry outcomes in the case group.

## Discussion

When comparing the spirometric lung function of Malawian children who had been admitted 7 years previously with an episode of SAM against “healthy” sibling and community controls, all children (cases and controls) were found to have significant reductions in spirometry as compared to international reference data. There were however no significant differences in spirometric lung function or oxygen saturation during an exercise test between SAM survivors and controls. In the sample as a whole, being HIV positive, of female sex, having less lean mass and having a history of TB were associated with lower spirometric z-scores. Although household smoke was not associated with lung function in the sample as a whole, when stratified by sex, we found that females exposed to indoor cooking smoke had a lower mean FEV_1_ z-score than unexposed females.

Deficits in spirometric z-scores compared to the international reference seen in all study groups likely reflects common environmental factors such as the high background levels of chronic undernutrition and general morbidity found throughout childhood in this region. A recent community survey in urban Malawian adults found the prevalence of spirometric restriction was 38.6% using NHANES (National Health and Nutrition Examination Survey) reference ranges (derived from a healthy Caucasian population in the USA) and 9.0% using local reference ranges [28]. Spirometric restriction was significantly associated with low BMI, which supports the theory that common environmental factors such as chronic undernutrition, combined with high population levels of biofuel exposure and HIV, may be sufficient to cause lung restriction, regardless of early SAM exposures.

Besides high levels of chronic undernutrition, it is important to consider the complex interaction of lung function, height, sitting height and leg length, as this could also diminish the effects of SAM in these results. Because sitting height as a percentage of standing height was on the hypothesised causal pathway between SAM and spirometry outcomes, our results were not adjusted for this variable. However, sitting height is an important consideration as spirometry z-scores are based on standing height. Although SAM survivors had a significantly lower HAZ, they had a similar sitting height as controls, which could have artificially “improved” their lung function z-scores and hence, potentially masked the long-term effects of SAM. In an attempt to unravel this, regression models including sitting height percentage and leg length were run but had virtually no effect on results; p-values remained insignificant for all spirometry outcomes.

Owing to this complication, it is important to emphasise that for their standing height*,* SAM survivors, compared to controls, have preserved lung function. Despite results from both animal models and some human studies in India, which suggest that postnatal malnutrition can cause qualitative changes in lung function beyond merely an effect on lung size [29–31], emerging evidence suggests that nutritional insults in childhood and/or consequential stunting results in smaller lungs that are not necessarily associated with restrictive or obstructive defects [32, 33]. This also seems to be true for our stunted SAM survivors, similar to results recently observed in a population of undernourished Indian children [32]. Another study hypothesised that although poor linear growth often does not affect lung function, poor weight gain does, possibly due to its negative effect on muscle function [34]. Our finding that lean mass was positively associated with FEV_1_ and FVC z-scores supports this hypothesis that weight, particularly muscle mass, may be more important for lung function than linear growth, particularly if weight loss has been substantial.

The finding that lung function in our cohort of SAM survivors appears preserved to the level of control children is particularly interesting considering recently published findings that survivors of SAM have multiple adverse long-term outcomes compared with controls, including low weight- and height-for-age, and weaker hand grip strength [18]. Such observations could be explained at least in part by the thrifty phenotype hypothesis of Hales and Barker [35, 36], which emphasises that poor growth is often associated with selective preservation of vital organs at the expense of other organs and tissues. This may apply to SAM survivors in whom limb growth was compromised during or after the episode of SAM, whereas sitting height and lung function appear preserved for the majority of survivors. This pattern of shorter legs but preserved torso height may allow lung function to be preserved by decreasing the “load” on the lungs, whose primary function is to keep the organs and tissues (especially the brain) supplied with oxygen (see load/capacity theory for more detail [37]). While the thrifty phenotype hypothesis emphasised “brain sparing” [38], the need for preserved lung function in order to support brain energy metabolism is a novel concept hypothesised by these results. It is possible that SAM survivors in this study have been successfully “thrifty” in leg growth, thereby preserving an important vital organ: the lungs.

Although these results suggest that lung function is unlikely to be a key area for intervention to curb post-SAM mortality, they do highlight some potentially “high risk” groups. One particularly susceptible population appears to be children with HIV. There is emerging evidence of an increased prevalence of chronic lung disease, including both obstructive and restrictive types, in HIV-positive adults [39, 40]. Despite 85% (62/73) of the HIV-positive children in this cohort reportedly taking antiretroviral therapy, their lung function was relatively impaired when compared to HIV-positive children. Very few studies have considered the effect of HIV on lung function in children; there is one recent study in India that also found significantly lower FEV_1_ and FVC in children aged 5–12 years with vertically transmitted HIV, the majority of whom were being administered antiretroviral therapy [41]. Repeat infections, such as TB, combined with lower WAZ and lower lean mass could all contribute to the reduced lung function in the HIV-positive group.

Besides HIV, females in this cohort also had significantly lower FEV_1_ and FVC z-scores compared with males. We hypothesised that this could be due to a deviation from the reference values because of delayed puberty in Malawian girls; however, the sex difference is still seen in young girls (<9 years), so this seems to be an unlikely explanation. The sex difference could also reflect differences in body composition or biofuel smoke exposure. Male patients in our cohort had significantly more lean mass than girls (based on BIA); therefore, differences in body composition may have contributed to the observed sex differences in spirometry. Although we did not find any overall association between spirometry outcomes and living with a tobacco smoker or cooking with solid fuel inside homes, differential exposure to cooking smoke between the sexes could also be a contributory factor, especially since biofuel smoke was associated with spirometry outcomes when our data were stratified by sex. However, we would have expected pollution exposure to result in an obstructive pattern rather than the restrictive pattern observed. Interventions such as low-pollution cook stoves, currently being evaluated in Malawi (www.capstudy.org/), could have particular benefits for high-risk groups.

### Limitations

Possible sources of bias in this study include survivor bias, selection bias with regard to controls, and observer bias. As 46% of the case group are known to have died since admission, survivor bias could have diminished the observed effects of SAM as only the “healthiest” with the fewest long-term impacts are likely to have survived. This survivor bias, as well as high rates of HIV and biomass smoke exposure, means that results may not be generalisable to all settings. In addition, the control group are not necessarily representative of the Malawian population as a whole, as they were recruited in the same communities as survivors of SAM. However, based on mean HAZ and WAZ from Malawi's 2015 DHS, our community controls appear similar to the general population (HAZ −1.3 *versus* −1.5 in DHS; WAZ −1.2 *versus* −0.8 in DHS) [42]. Selection bias could have also affected the community control group if those who chose to participate differed systematically from those who declined. This could not be assessed as data were not available on those who declined or dropped-out of the study. Lastly, observer bias could have influenced the results as spirometry is an effort-dependent test, and operators were not blinded to the case/control status of the participants due to risk of data-entry errors. However, this is in part mitigated by the fact that those conducting quality control and selection of final spirometry traces for analysis were blinded.

Other limitations of this study include the lack of information on some potential confounders such as the accurate birth weight data. Birth weight is associated with long-term adverse effects on lung function [2] and is also associated with a higher risk of SAM in infancy [43]. However, using the mother's estimate as to whether the child was “small” or “normal” at birth, we found no association with lung function. Finally, it is important to note that our results may be affected by the length of follow-up. It could be that small inter-group differences are not yet clinically apparent. Evidence of “lung sparing” growth could indicate phenotype adaptation, which might not have apparent adverse consequences until much later in life [44]. Future follow-up in adulthood could address this.

### Conclusion

Contrary to our initial hypothesis, we found no significant long-term impact of SAM on lung function in surviving children. This could be due to “thrifty” or “lung-sparing” growth preserving sitting height and lung function at the expense of limb length. HIV, despite treatment, was associated with adverse effects on spirometry outcomes, even at these young ages. Females also had significantly poorer lung function than males in this cohort: this could reflect differences in body composition or cooking smoke exposure. These groups could be considered high-risk populations in intervention packages seeking to improve lung function both in survivors of SAM and in the general population.

## Supplementary material

10.1183/13993003.01301-2016.Supp1 **Please note:** supplementary material is not edited by the Editorial Office, and is uploaded as it has been supplied by the author.
Supplementary material ERJ-01301-2016_Supplement

## Disclosures

10.1183/13993003.01301-2016.Supp2 J.C. Wells ERJ-01301-2016_Wells
