# Supplementary material for: Long-term effects of severe acute malnutrition on lung function in Malawian children: a cohort study
Source: Eur Respir J. 2017 Apr 6;49(4):1601301. doi: 10.1183/13993003.01301-2016 (PMC5540677; doi:10.1183/13993003.01301-2016)
Supplement: Supplementary file 1 [file ERJ-01301-2016_Supplement.pdf]

Supplement: Glossary of common nutritional terms

- **BIA:** Bioelectrical Impedance Analysis (BIA) is a technique used for measuring body composition. It uses the principle that electric current flows at different rates through the body depending upon its composition of fat and water. It therefore uses measures of Resistance and Reactance (together known as Impedance) along with height to estimate total body water, fat mass and lean mass.
- **DOHaD:** The developmental origins of health and disease theory (DOHaD) is a multi-disciplinary field of research that examines how environmental factors during the phase of developmental plasticity (i.e. prenatal or early postnatal life) interact with genotypic variation to change the capacity of the organism to cope with is environment in later life, often resulting in adult diseases.
- **Lean mass index (LMI):** Lean mass, usually calculated from BIA readings, can be converted to LMI by diving it by the square of height (lean mass (kg) / height $(m)^2$). Lean mass calculated from BIA is not adjusted for body size, hence LMI provide a discrete measure of *relative* lean mass, expressed in the same kg/m2 units as BMI. When an appropriate conversion equation is not available to convert BIA readings into lean mass (kg), a useful "LMI equivalent" can be calculated simply by dividing 1/Resistance (R).
- **Oedema:** Bilateral oedema, also known as Kwashiorkor, is a form of severe acute malnutrition (SAM) characterised by too much fluid in body tissues which causes swelling under the skin (called oedema). It usually begins in the legs, but can involve the whole body, including the face. An enlarged abdomen, irritability, yellow hair, drowsiness and patches of inflamed or peeling skin may also be present. If the condition is left untreated, it can be fatal. The word derives from the Ghanaian language meaning "weaning sickness" as it often presents in children around the age of weaning. The exact physiological causes behind kwashiorkor are still unknown.
- **SAM:** Severe acute malnutrition is defined by a very low weight for height (below -3z scores of the median WHO growth standards); by low mid-upper arm circumference (MUAC) (<11.5cm); by visible severe wasting, or by the presence of nutritional oedema**.** SAM is the most dangerous form of malnutrition; if left untreated, it can result in death. Until 2006, the recommendation was to refer these children to hospital to receive therapeutic diets along with medical care. Treatment recommendations have now changed following the advent of "ready to use therapeutic foods" (RUTF) which allows the management of SAM in the community for those children above the age of 6 months and without medical complications.
- **Stunting:** stunting is defined as low height for age. Using a WHO international growth reference for a "normal" height for age, it is possible to classify whether and by how much a child is stunted using z-scores. Zero z-scores is the global norm therefore anything below zero is less than the global norm. Generally, a z-score less than -2 is classified as stunted. A z score less than -3 is severely stunted.
- **Wasting:** wasting is a form of severe acute malnutrition; it can also be referred to as Marasmus. Wasting is characterised by a loss of muscle mass, usually assessed by anthropometry; weight-for-height and MUAC are the measures recommended by the WHO.
- **Underweight:** underweight is usually defined has having a low weight for age. Using a WHO international growth reference for a "normal" weight for age, it is possible to classify whether and by how much a child is underweight using z-scores. Zero z-scores is the global norm therefore anything below zero is less than the global norm. Generally, a z-score less than -2 is classified as underweight.

Table S1: Result of Simple and Multivariable regression analysis for iStep exercise test outcomes

	Cases	Sibling			Community		
**iStep outcomes (n=497)**	**Mean** (SD)	**Mean** (SD)	**Unadjusted difference** (95% CI)	**Adjusted difference** (95% CI)	**Mean** (SD)	**Unadjusted difference** (95% CI)	**Adjusted difference** (95% CI)
**Baseline HR (BPM)**	99·9 (15·1)	98·6 (15·4)	-1·29 (-4·5,1·9)	-0·23 (-3·6,3·2)	99·1 (14·4)	-0·76 (-4·1,2·6)	-1·0 (-4·6,2·5)
**Highest HR (BPM)**	154·8 (21·9)	156·7 (20·9)	1·82 (-2·7,6·3)	-0·47 (-5·3,4·4)	159·5 (19·5)	4·22 (-0·5,9·0)	4·24 (-0·9,9·3)
**Resting O_2 saturation %**	97·2 (3·2)	97·2 (3·2)	0·03 (-0·5, 0·5)	0·13 (-0·5, 0·7)	97·7 (2·0)	0·46 (-0·1, 1·0)	0·54 (-0·1, 1·2)
**Lowest O_2 saturation %**	93·4 (4·9)	93·7 (4·9)	0·37 (-0·6, 1·4)	0·73 (-0·4, 1·8)	93·8 (3·8)	0·45 (-0·6, 1·5)	0·29 (-0·9, 1·4)

Adjusted difference includes age, sex, HIV status and SEC in the regression model. HR = heart rate. No significant differences were observed as reflected by 95% Confidence intervals (CI) all of which encompass zero.
